# CCR5 Blockade in Inflammatory PML and PML-IRIS Associated With Chronic Inflammatory Diseases' Treatments

**DOI:** 10.1212/NXI.0000000000001097

**Published:** 2021-11-02

**Authors:** Raphael Bernard-Valnet, Xavier Moisset, Nicolas Maubeuge, Mathilde Lefebvre, Jean-Christophe Ouallet, Mathilde Roumier, Christine Lebrun-Frenay, Jonathan Ciron, Damien Biotti, Pierre Clavelou, Bertrand Godeau, Renaud A. Du Pasquier, Guillaume Martin-Blondel

**Affiliations:** From the Service of Neurology (R.B.-V., R.A.D.P.), Department of Clinical Neurosciences, Lausanne University Hospital (Centre Hospitalier Universitaire Vaudois) and Lausanne University, Switzerland; Université Clermont Auvergne (X.M., P.C.), CHU de Clermont-Ferrand, Inserm, Neuro-Dol, ; Department of Neurology (N.M.), CHU de Poitiers, Hôpital La Milétrie; Department of Infectious Diseases (M.L., G.M.-B.), Toulouse University Hospital; Service de Neurologie, Pôle des Neurosciences Cliniques (J.-C.O.), CHU de Bordeaux Pellegrin Tripode; Service de Médecine Interne (M.R., B.G.), CHU Henri Mondor, Créteil; CRCSEP Nice (C.L.-F.), CHU de Nice, Université Nice Côte D'Azur, UR2CA-URRIS, Neurologie Pasteur 2; Department of Neurosciences (J.C.,D.B.), Toulouse University Hospital, France.

## Abstract

**Background and Objectives:**

Progressive multifocal leukoencephalopathy (PML) is a disabling neurologic disorder resulting from the infection of the CNS by JC polyomavirus in immunocompromised individuals. For the last 2 decades, increasing use of immunotherapies leads to iatrogenic PML. Iatrogenic PML is often associated with signs of inflammation at onset (inflammatory PML) and/or after treatment withdrawal immune reconstitution inflammatory syndrome (PML-IRIS). Although immune reconstitution is a key element for viral clearance, it may also be harmful and induce clinical worsening. A C-C chemokine receptor type 5 (CCR5) antagonist (maraviroc) has been proposed to prevent and/or limit the deleterious immune responses underlying PML-IRIS. However, the data to support its use remain scarce and disputed.

**Methods:**

We conducted a multicenter retrospective cohort study at 8 university hospitals in France and Switzerland by collecting clinical, biological, and radiologic data of patients who developed inflammatory PML (iPML) or PML-IRIS related to immunosuppressive therapies used for chronic inflammatory diseases between 2010 and 2020. We added to this cohort, a meta-analysis of individual case reports of patients with iPML/PML-IRIS treated with maraviroc published up to 2021.

**Results:**

Overall, 27 cases were identified in the cohort and 9 from the literature. Among them, 27 met the inclusion criteria: 16 treated with maraviroc and 11 with standard of care (including corticosteroids use). Most cases were related to MS (92.6%) and natalizumab (88%). Inflammatory features (iPML) were present at onset in 12 patients (44.4%), and most patients (92.6%) received corticosteroids within the course of PML. Aggravation due to PML-IRIS was not prevented by maraviroc compared with patients who received only corticosteroids (adjusted odds ratio: 0.408, 95% CI: 0.06–2.63). Similarly, maraviroc did not influence time to clinical worsening due to PML-IRIS (adjusted hazard ratio = 0.529, 95% CI: 0.14–2.0) or disability at the last follow-up (adjusted odds ratio: 2, 95% CI: 0.23–17.3).

**Discussion:**

The use of CCR5 blockade did not help to keep deleterious immune reconstitution in check even when associated with corticosteroids. Despite maraviroc's reassuring safety profile, this study does not support its use in iPML/PML-IRIS.

**Classification of Evidence:**

This study provides Class IV evidence showing that adding maraviroc to the management of iatrogenic iPML/PML-IRIS does not improve the outcome.

Progressive multifocal leukoencephalopathy (PML) is a rare neurologic disorder caused by the reactivation of JC virus in immunocompromised hosts, which leads to lytic infection of oligodendrocytes.^1^ The proportion of non–HIV-infected patients developing iatrogenic PML is increasing as a result of the use of biological immunosuppressive/modulatory agents in chronic inflammatory or rheumatologic diseases, especially in patients with multiple sclerosis (MS) who were treated with natalizumab.^2,3^ Recovery of JC virus–specific immune responses by withdrawing immunosuppressive therapies remains the only possibility to treat PML.^4^ However, immune restoration is not always beneficial, and a variable proportion of patients with PML worsen because of severe neuroinflammation, which results from immune reconstitution inflammatory syndrome (PML-IRIS). PML-IRIS is thought to arise from an excessive protective immune response against pathogen-derived antigens that causes disproportionate tissue damage to the host.^4^ CD8^+^ T cells are abundant in PML-IRIS lesions and are considered to be the probable drivers of PML-IRIS.^5,6^ Then, management of PML-IRIS relies on corticosteroids in most severe cases.^7^ Systematic use of preemptive corticosteroid therapy has been proposed for patients with MS who develop PML during natalizumab therapy, which is usually associated with inflammatory features from PML onset, to prevent the development of PML-IRIS after natalizumab withdrawal.^8^ However, steroids have a profound impact on the JC virus (JCV)-specific T-cell response and might compromise the control of JCV replication.^9^ Therapeutic strategies that selectively alleviate inflammation without hampering immune restoration are therefore desirable. Because the CCR5/CCL5 axis is implicated in T-cell activation and leukocyte trafficking to the brain in the setting of neurotropic infections and experimental models of MS, as well as in human MS, it was hypothesized that C-C chemokine receptor type 5 (CCR5) antagonists such as maraviroc, a noncompetitive CCR5 antagonist approved in the HIV armamentarium, might prevent and/or treat the deleterious inflammatory reaction that occurs during immune recovery in patients with PML, by interfering with the activation and/or migration of CCR5- expressing activated CD8^+^ T cells.^10^ However, evidence supporting the use of maraviroc remains scarce with few cases reported in the literature.^11-16^ Our aim was to assess whether maraviroc influences the prognosis of iatrogenic PML with inflammatory features and PML-IRIS in patients with chronic inflammatory or rheumatologic diseases.

## Methods

### Study Design and Patient Selection

We conducted a retrospective multicenter cohort study through a network of 8 university hospitals in France and Switzerland. Inclusion criteria were patients with chronic inflammatory or rheumatologic diseases receiving biological immunosuppressive/modulatory agents who developed definite, probable, or possible PML according to the American Academy of Neurology criteria^17^ between 2010 and 2020 and PML displaying inflammatory features such as enhancement of PML lesions after gadolinium injection, edema, or mass effect that are not usually associated with classic PML. Two distinct settings were individualized: inflammatory PML (iPML) when inflammatory features were present at PML onset before cessation of the immunosuppressive agent as defined previously^18^ and PML-IRIS when a patient with PML clinically worsened after cessation of the immunosuppressive agent, while inflammatory features appeared or increased on the brain MRI. Data were collected in each center by filling anonymous case report forms and centralized in Toulouse. Outcomes were determined by the physician in charge. A literature review was also performed searching for case reports/series of patients with PML treated with maraviroc up to April 1, 2021 (MeSH terms: [progressive multifocal leukoencephalopathy] AND [maraviroc]; [immune reconstitution inflammatory syndrome] AND [maraviroc]).

### Treatment Groups

Patients who received maraviroc continuously for more than 2 weeks were included in the maraviroc group. Patients who had never received maraviroc were assigned to the control group. Patients who received maraviroc before PML-IRIS were considered as a preventive scheme and patients who received maraviroc after PML-IRIS onset as therapeutic scheme.

### Outcomes Measures

The primary outcome was neurologic worsening under treatment defined by worsening of the modified Rankin Scale (mRS) score ≥1 point. Exploratory variables were the interval elapsed between maraviroc introduction and clinical worsening under treatment, PML-IRIS occurrence, PML-IRIS duration (defined as the time elapsed between clinical deterioration due to PML-IRIS and the first clinical follow-up with stable/improving deficit and reduction of gadolinium-enhancing lesions on MRI), disability at 12 months (measured by the mRS score) and at last follow-up, and increase in disability (defined as an increase in the mRS score ≥2 before and 12 months after PML).

### Statistical Analysis

According to the Strengthening the Reporting of Observational Studies in Epidemiology (STROBE) guidelines, patient characteristics are expressed as median (interquartile range [IQR]) for continuous variables and n (%) for categorical variables. Groups were first compared using Fisher exact tests (categorical variables) and Mann Whitney *U* tests (continuous variables). Furthermore, a binomial logistic regression was run to evaluate the effect of maraviroc on clinical worsening under treatment, PML-IRIS occurrence, and increase in disability. A Cox hazard model was used for the analysis of time to worsening. Both models were adjusted for sex, age, baseline mRS score, and multilobar presentation. Analysis was conducted using SPSS Statistics 27.0 (IBM SPSS Statistics for Windows, Armonk, NY).

### Ethical Approval

This study was approved by an institutional review board (RnIPH 2021-35), in accordance with the French data protection authority (MR004, Commission Nationale de l'Informatique et des Libertés, CNIL number 2206723v0). Consultation of an ethics committee was not required as this was a noninterventional study, which does not fall under the French Jardé law. According to the French law on ethics, patients were informed that their anonymized data would be used in the study and for publication. Their nonopposition to the use and publication of the data was collected. For Switzerland, all patients were included in the COOLIN BRAIN project approved by the local ethic committee (CER-VD—approval number: 2018-01622).

### Data Availability

The anonymized data that support the findings of this study are available from the corresponding author on reasonable request for non-commercial purposes.

## Results

### Baseline Characteristics of Progressive Multifocal Leukoencephalopathy

**Figure 1:**
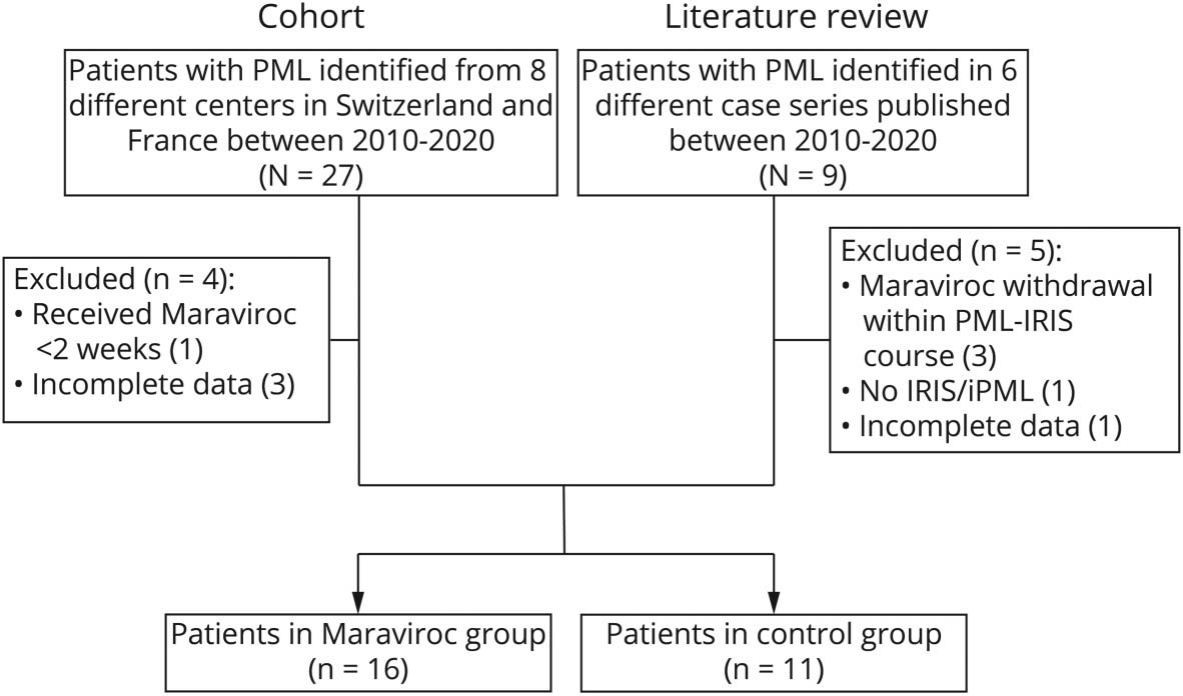
Flowchart

Among 27 patients with iatrogenic iPML or PML-IRIS retrospectively identified in the participating centers, 23 were kept for final analysis, of whom 12 received maraviroc. Three patients were excluded because of missing data and 1 because the patient received maraviroc for less than 1 week. Furthermore, 9 cases of patients with iPML or PML-IRIS who received maraviroc were retrieved from the literature and 4 were included in our analysis (flowchart, Figure 1).^11-16^ Therefore 27 iPML or PML-IRIS cases were included in this study, of whom 16 received maraviroc, and 11 did not and served as controls.

Patients and PML characteristics in both groups are presented in Table 1. Patients with PML were mainly women (19/27, 70.4%) with a median age at PML onset of 43 years (IQR 7). All were iatrogenic PML, mostly related to natalizumab (24/27, 88.9%) and MS (25/27, 92.6%). Two patients received mycophenolate mofetil/corticosteroids and corticosteroids, respectively, for dermatomyositis and rheumatoid arthritis. PML was definite in 22/27 patients (81.5%), probable in 3/27 patients (11.1%), and possible in 2/27 patients (7.4%). At PML onset, brain MRI showed a multilobar involvement in 19/27 patients (70.4%). Inflammatory features with gadolinium-enhancing lesions and/or mass effect were present in, respectively, 11/27 patients (40.7%) and 3/27 patients (11.1%). Median CSF JC viral load was 868 copies/mL (IQR 3,607 copies/mL). Immunosuppressive/modulatory agents were interrupted in 27/27 (100%) cases, and plasma exchanges for natalizumab removal were performed in 20/24 patients (83.4%). Finally, 12/27 patients (44.4%) with contrast-enhancing lesions and/or edema were defined as having iPML at baseline, and 23/27 patients (85.2%) experienced clinical worsening related to PML-IRIS after cessation of immunosuppressive drugs within a median duration of 4 weeks (IQR 4.5 weeks). The mean overall follow-up after PML onset was 23 months (IQR 34 months). There was no difference between the 2 groups (Table 1).

**Table 1:**
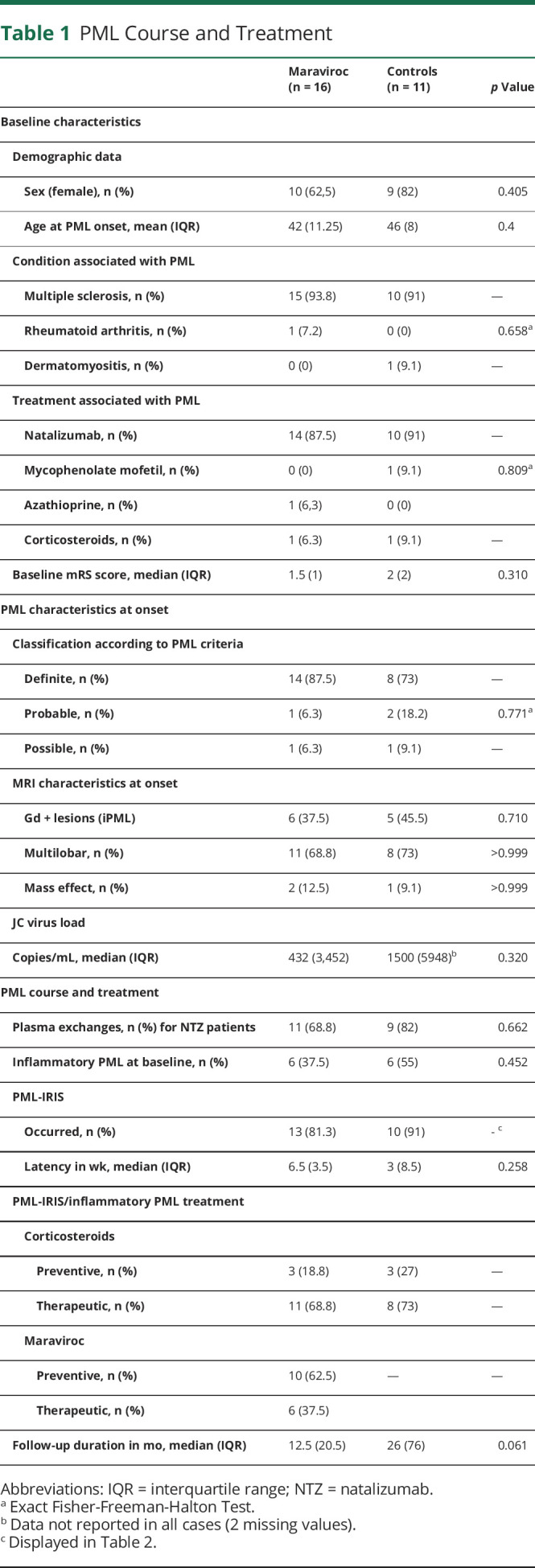
PML Course and Treatment

### Maraviroc Use in Inflammatory Progressive Multifocal Leukoencephalopathy and Progressive Multifocal Leukoencephalopathy Immune Reconstitution Inflammatory Syndrome

As previously stated, 16/27 patients (59.2%) received maraviroc for the management of iPML and/or PML-IRIS; 10 of 16 patients (67.5%) received maraviroc in the hope of preventing clinical worsening due to PML-IRIS (preventive), and 6/16 (37.5%) received maraviroc at PML-IRIS onset to alleviate immune responses affecting the brain (therapeutic). The median time from PML diagnosis to maraviroc introduction was 10.5 days (IQR 26.75 days). Steroids were concomitantly administered in 14/16 (87.5%) patients treated with maraviroc and 11/11 (100%) patients not treated with maraviroc.

**Figure 2:**
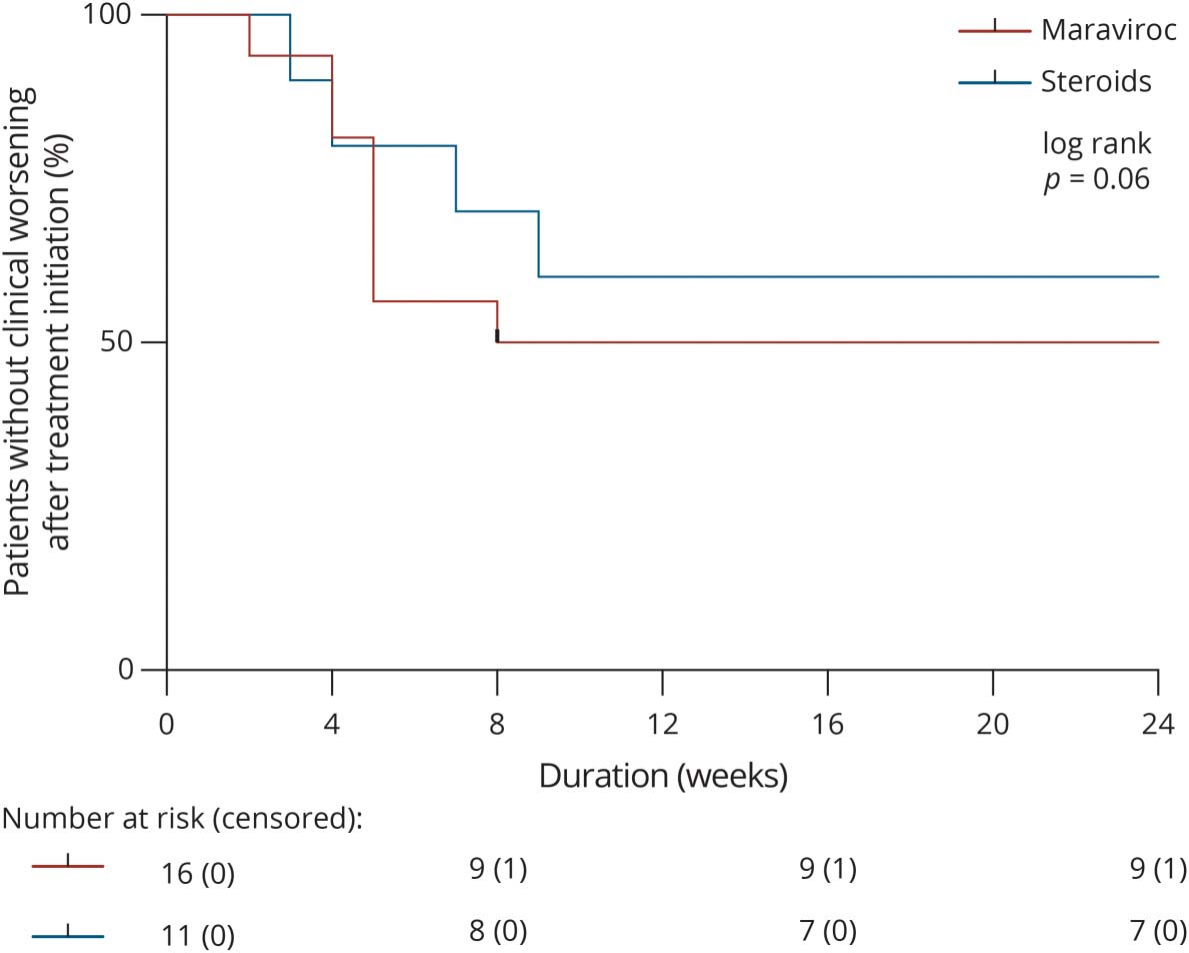
Probability of Survival Without Clinical Worsening After Treatment Introduction The graph depicts the proportion of patients free of clinical worsening following maraviroc (red line) or steroid (blue line) initiation. Ticks represent censored data. Above the graph are displayed the number at risk (and censored) in each group.

Next, we evaluated whether maraviroc improved the overall outcome of PML regardless of the setting in which it was prescribed (Table 2). Maraviroc did not mitigate the occurrence of clinical worsening after treatment initiation (8/16 vs 4/11, *p* = 0.696), nor did it lengthen the interval before clinical worsening (median: 5 vs 5.5 weeks, *p* = 0.728) (Figure 2). Despite the preventive use of maraviroc in half of the cases, the incidence of PML-IRIS (13/16 vs 10/11, *p* = 0.624) and the latency from treatment withdrawal to PML-IRIS (median: 6.5 vs 3 weeks, *p* = 0.258) were similar in the 2 groups. Similarly, the use of maraviroc during PML-IRIS did not affect the duration of IRIS (median: 9.5 vs 9 weeks, *p* = 0.943).

**Table 2:**
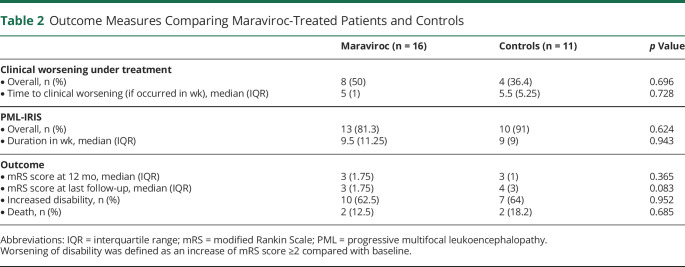
Outcome Measures Comparing Maraviroc-Treated Patients and Controls

Regarding long-term prognosis, maraviroc did not improve neurologic status at 12 months (median mRS score: 3 vs 3, *p* = 0.365) or at the last follow-up (median mRS score: 3 vs 4, *p* = 0.083). Furthermore, most patients had a significant increase in disability due to PML that did not differ between the 2 groups (10/16 vs 7/11, *p* = 0.952). Three patients died, 2 in the maraviroc group (2/16, 12.5%), 1 from aspiration pneumonia and 1 from PML progression, and 2 in the control group (2/11, 18.4%) from status epilepticus related to PML sequelae and IRIS aggravation, respectively.

We analyzed these outcomes using a logistic regression model adjusted for sex, age, baseline mRS score, and multifocal involvement. There was no difference between groups when assessing the occurrence of clinical worsening after treatment initiation (unadjusted odds ratio: 0.571, 95% CI: 0.12–2.8, adjusted odds ratio: 0.408, 95% CI: 0.06–2.63), the occurrence of PML-IRIS (adjusted odds ratio: 2.3, 95% CI: 0.2–25.6, adjusted odds ratio: 1.2, 95% CI: 0.07–19.6), or overall increased disability (unadjusted odds ratio: 1.05, 95% CI: 0.2–5.2, adjusted odds ratio: 2, 95% CI: 0.23–17.3). Similarly, using a Cox Hazard model corrected for the same variables, maraviroc did not affect time to neurologic worsening under treatment (unadjusted hazard ratio = 0.658, 95% CI: 0.2–2.2, adjusted hazard ratio = 0.529, 95% CI: 0.14–2.0)

Despite low numbers of patient, we then tried to decipher if timing of introduction of maraviroc could affect the outcome. Early maraviroc initiation within 7 days after diagnosis of PML occurred in 8/16 patients (50%) and did not affect the 12-month outcome (median mRS score 3 vs 3, *p* = 0.442). Similarly, we hypothesized that maraviroc introduction in patients presenting already signs of iPML/PML-IRIS could be too late to halt the process. However, outcome was not influenced in the 8/16 patients (50%) who initiated maraviroc while they had no gadolinium-enhanced lesions (median mRS score: 3 vs 3, *p* = 0.959). Regarding the safety of maraviroc use, no significant adverse events related to this treatment were reported.

## Discussion

In this multicenter retrospective cohort and review of the literature, we investigated whether the use of CCR5 antagonists influences the outcome of iatrogenic iPML/PML-IRIS related to immunosuppressive agents for the treatment of chronic inflammatory diseases. We were not able to show that maraviroc improves the outcome of iPML/PML-IRIS on all the variables investigated.

Correlative evidence implicating the CCR5–CCL3/CCL5 axis in MS, Rasmussen encephalitis, and infectious diseases, such as cerebral malaria and HIV-associated neurocognitive disorders, led to the hypothesis that CCR5 blockade might provide neuroprotection in settings in which CCR5 contributes to deleterious neuroinflammation, particularly in diseases in which CD8^+^ T cells play a pivotal role.^10^ It has been shown that most brain lesion–infiltrating CD8^+^ T cells express CCR5 in HIV-infected and non–HIV-infected patients developing inflammatory forms of PML.^19,20^ Thus, the use of CCR5 antagonists, such as maraviroc, which have already been developed as entry inhibitors of HIV-1, may appear as an attractive alternative to steroids to prevent PML-IRIS. Indeed, if steroids remain a cornerstone of PML-IRIS treatment, as demonstrated by our study (25/27 of our patients received steroids for the management of iPML/PML-IRIS), their role remains controversial, especially as preemptive treatment, as steroids may affect anti-JCV cellular immune responses broadly and induce a remanent suppression.^9,21^ This rationale was further substantiated by occasional case reports from the fields of HIV, and later MS, showing good outcomes after maraviroc use in PML-IRIS.^14,19,22^ One of them described a patient with MS developing natalizumab-associated inflammatory PML and showed that maraviroc exhibited preventive and curative properties.^14^ The use of maraviroc was strikingly associated with a selective reduction in the levels of CCR5^+^ T cells in the CSF, suggesting that CCR5 antagonists limit immune cell trafficking into the CNS in vivo. However, the beneficial impact of maraviroc in PML-IRIS management was not supported by other case reports and case series in natalizumab-associated PML.^12^ Recently, a retrospective cohort of 34 patients with HIV-associated PML found no clinically relevant benefit of maraviroc as part of antiretroviral therapy on overall survival.^23^ More generally, 2 controlled clinical trials showed that maraviroc added to standard antiretroviral therapy for advanced HIV did not mitigate the risk of developing IRIS.^24,25^ We may, however, point that in our study, the overall outcome at last follow-up showed a tendency to a lower degree of disability in the maraviroc group. This clinically meaningful difference may be due in the control group to a longer follow-up and to the late death (>12 months) of 1 patient due to status epilepticus on PML lesions.

A few hypotheses may be drawn to explain the lack of efficacy of CCR5 blockade to prevent and treat PML-IRIS. First, CCR5 is widely expressed on the cell surface of activated CD4^+^ or CD8^+^ T cells.^26^ The wide CCR5 expression on brain lesion-infiltrating CD8^+^ T cells demonstrated in inflammatory PML could therefore only reflect the activation of effector T cells, without indicating an implication of the CCR5–CCL3/CCL5 axis in migration and homing of CD8^+^ T cells to the CNS in PML-IRIS. Second, regulatory T cells express CCR5. CCR5 blockade might therefore also inhibit CCR5-dependent migration of regulatory T cells to inflamed tissues and, consequently, disrupt local immune regulation, abrogating a potential benefit of the limitation of effector T-cell homing. Third, maraviroc is thought to have good CNS penetration (CNS penetration-effectiveness of 3) based on CSF concentrations found in treated patients with HIV.^27^ However, mitigating T-cell pathogenicity might require higher CNS concentrations for CCR5 blockade than those needed to control HIV replication as part of highly active antiretroviral therapy. Finally, initiation of maraviroc while inflammatory features already exist, and pathogenic T cells have already penetrated the brain parenchyma, may simply be too late to expect a potential benefit. Further studies could be valuable to assess CCR5 chemokines CCL3, CCL4, and CCL5 in the periphery and in the CSF in the context of classical PML and in inflammatory PML, as well as the effect of CCR5 blockade on the proportion and number of CCR5-expressing T cells in the CSF and in brain tissue.

We may point out several limitations in this study inherent to orphan diseases. First, the analysis has been performed on limited number of cases. We were unable to calculate a priori statistical power and sample size needed to reject type 2 error. Then, we cannot exclude a mild effect of maraviroc to prevent aggravation due to PML-IRIS. Furthermore, it is important to stress that the wide immunomodulating effect of corticosteroids used in most patients is a major confounder that could have masked a potential beneficial effect of maraviroc. However, most patients in the maraviroc group (69%) received steroids only in case of full blown PML-IRIS. Among limitations, we should also acknowledge that retrospective design may affect data collection. Similarly, in absence of standardized consensus in PML management, the timing of introduction of steroids and maraviroc was not homogeneous among cases. These differences in management represent a confounding factor difficult to be addressed on such a small sample size. Finally, most cases were collected by neurologists, which could preclude the generalizability of these results to all PML associated with chronic inflammatory diseases treatments.

The past 25 years in the field of PML have provided repeated examples that the benefits of potential drugs have often been overstated based on isolated case reports or small series, as is the case for maraviroc. Our study that failed to show a beneficial impact of maraviroc in the management of iPML/PML-IRIS has to be confirmed on a larger data set.

## Glossary

CCR5: C-C chemokine receptor type 5
iPML: inflammatory PML
JCV: JC virus
mRS: modified Rankin Scale
MS: multiple sclerosis
PML: progressive multifocal leukoencephalopathy
PML-IRIS: progressive multifocal leukoencephalopathy immune reconstitution inflammatory syndrome
